# The Student's "Reckoning Day"

**Published:** 1889-03-09

**Authors:** 


### The Hospital

A Weekly Institutional Journal of

Science, Medicine, Nursing, and Philanthropy.

*For Hospitals, Asylums, Medical Practitioners, Students, Nurses, Families, Parishes, Congregations, and the Charitable Public.*

Vol. V. No. 128.] [March 9, 1889.

### The Student's "Reckoning Day."

The student with a sense of humour will naturally ask what particular "reckoning day" is meant by the title of this article? He is conscious that a good many reckoning days are looming in the distance, if not for himself, at least for a considerable proportion of the men of his acquaintance. The medical student will not be too exalted to think even of his landlady in this connexion: whilst his tailor and his bookseller may also shadow themselves forth on the horizon of the near or distant future. But these are feeble and insignificant anticipations compared with the feeling as of irrestible destiny which lays hold of the second year's man's heart as he thinks of the day of his "first professional examination." The battle has got to be faced! What is the state of his courage? Above all, what is the condition of his training and of his weapons? For that, with the young Briton, medical or otherwise, is generally the vital question of the moment. Courage as a rule is not wanting, even in the man who knows himself to be as unequal to the ordeal before him as a cow is to leaping over a five-barred gate. Such courage ought perhaps to be called by another name. Possibly even "impudence" would not be too strong for the occasion. Be that as it may, the question of questions for students at examinations is that of "weapons and training."

By "weapons" we mean knowledge; and by "training" the art of using it. The knowledge required by a medical student, even for his first professional examination, is peculiar in kind and considerable in quantity. The training needed to enable him to use his knowledge with judgment and measure is, or ought to be, of the most skilful and efficient character. The knowledge may be obtained from books; the training can only be acquired by constant contact with and help from competent trainers. In anticipation of the Spring Examinations which are approaching at so many medical boards, we desire to enter a plea on behalf of the student. Experience justifies the assertion that a large majority of medical students are sincerely desirous of passing their examinations creditably, and are quite willing to work hard to that end. It may be affirmed, with equal emphasis and unquestionable truth, that the help they receive from very many of their teachers is of the poorest, feeblest, and most unintelligent kind. Take the two subjects of Chemistry and Anatomy, both of which are subjects of examination early in the student's career. In teaching chemistry the lecturer lectures an hour a day for five or six months in the year to a class of a hundred young men, more or less. For three months a "practical chemistry" class is held, and batches of youths are carried at full gallop through a few stock chemical tests every day. And thus chemistry is "taught." To the majority of these young men the lecture by the professor is as so much Hebrew or Chinese, because he talks about substances and modifications with which they are as unfamiliar as they are with Descartes' Philosophy or Ricardo's Political Economy. To the larger number of the students "words" and not "things" are the "counters" with which the lecturer is dealing. But chemistry is eminently a science of "things," and in no sense of abstractions. The student thus gets the idea that what he requires is to be familiar with his text-book and his lecturer's peculiar way of looking at chemistry questions, and that then he will be all right for his examination. And his judgment proves to be correct. If on examination he is found familiar with the statements of any recognised text-book he is carried through without difficulty and with flying colours. But he may know nothing whatever of chemistry all the same. In proof whereof it is only necessary to ask the question, "How much does the average medical man who has been ten years in practice know even of the merest elements of chemical science?" Practically nothing! The lecturer and the examiner, instead of the one of them training the man thoroughly in so much of the science as he might reasonably be expected to master in the time allotted by the curriculum to the subject, and the other in discovering how much the pupil had made himself intelligently and comprehensively familiar with, have wasted their own time, and the student's time and money too, in doing him more harm than good. The one of them has pretended to "teach" him, and the other has pretended to "examine" him, and has labelled him "taught, examined and passed." Thus the man walks out of his college a living "fraud," innocent enough so far as his morals are concerned, but highly dangerous to the public.

The teaching of Anatomy is carried on in a very similar way, except that the student instead of spending an hour a day for three months, as in so-called "practical chemistry," usually spends two or three hours a day for two or three years in the dissecting-room. But what we insist upon is that he is not "taught" in any sense as he ought to be taught, but is to a very large extent left to pilot his way through a most difficult region by the very poor aid of dissecting books. Neither chemistry nor anatomy can be taught by lectures, nor even by "demonstrations." They can only be taught by strenuous personal labour on the part of the teacher, and by daily personal contact between teacher and pupil. It is not too much to say that the methods of instruction in many of our medical schools are at least a quarter of a century behind the times. Even the very Sunday Schools in many places are far ahead of them. In the latter separate class-rooms and separate teachers are provided for all except the very youngest, and few classes are allowed to consist of more than a dozen individuals. But what is the case in some well-known medical schools? A single class will consist of a hundred, of two hundred, or even of three hundred members and upwards. Let it be remembered too that such subjects as anatomy and chemistry are as new and foreign to most of the students as are the elements of religion and Biblical knowledge to the average child of seven. Will any rational man call this "teaching"? A small class, a separate room, a competent teacher for each class, the daily manipulation in turn by each student of every detail of his subject, not only under the eye but under the direct personal guidance of his teacher—these are absolutely indispensable to the efficient teaching of the sciences that are required for the knowledge and practice of the medical art. There are many men in medical practice to-day who are no more worthy of being called "skilled practitioners" than they are of being designated "great sculptors." Their ignorance and incompetence are fruitful sources of continued illness on the part of their patients, and not seldom are the direct causes of death.

Now where does the fault lie—with the teachers or with the students? We say emphatically with the teachers: with the teachers and with the system, for which the teachers are directly responsible. The teachers know that their system is bad; or if they do not they ought not to be teachers. They know very well that the average results produced are poor and feeble in an indescribable degree. Probably familiarity with such results may blind some of them to their real character; but every teacher in a medical school who is possessed of the smallest modicum of true teaching genius must feel that, tried by any true standard of intelligent merit, however modest, large numbers of his pupils would make displays of themselves which would be ludicrous in their utter incompetency. The students are very little, if at all, to be blamed. The problem they are asked to solve in their four years' curriculum is absolutely new to them. The vast continent of knowledge through which they have to pass consists entirely of foreign countries. They have no idea at the outset of its measureless extent, its natural features, its human inhabitants, its fauna, its flora, or its many and varying climates. They do not know what it is essential they should learn on their first exploration or what they may with safety leave for future journeys. To them in their scientific childhood an elephant is as important as a man, and even more important, because he is so very much larger. Months may be wasted in the pursuit of what is merely trifling or curious, whilst that which is essential may be neither understood nor even noticed. What then is the one condition of pleasant and profitable travelling? A competent, willing, cheerful, and enthusiastic guide. A teacher who will be always at hand, who is familiar with the way, and with everything that is to be met with on the way. One in a hundred among medical teachers is such a guide. Too many of the other ninety-nine are of the "Cathedral verger type," who know a few leading facts by heart, and repeat them so often that they cease to have the least spark of meaning either for themselves or for anybody else. If medical students are to meet their "Day of Reckoning" with cheerful confidence they must insist upon a sufficiency of competent teachers. They must insist, and insist, and insist again until they get them.

